# A Complex Case of Acute Liver Failure Following Idiosyncratic Drug-Induced Liver Injury, Potentiated by Herpes Simplex Virus Infection

**DOI:** 10.7759/cureus.74955

**Published:** 2024-12-02

**Authors:** Ana Raquel Soares, Pedro Fiúza, João Rodrigues, Marta Guisado Orantos, Paula Nascimento

**Affiliations:** 1 Internal Medicine, Unidade Local de Saúde de São José, Lisbon, PRT; 2 Pulmonology, Unidade Local de Saúde de São José, Lisbon, PRT

**Keywords:** acute liver failure, drug-induced liver injury, hepatic encephalopathy, herpes simplex virus, nintedanib

## Abstract

Acute liver failure (ALF) is a rare, life-threatening condition that may be secondary to drug-induced liver injury (DILI) and certain viral infections.

We present the case of a 73-year-old male with a history of fibrotic hypersensitivity pneumonitis with a progressive phenotype, type 2 diabetes mellitus, hypertension, and hyperlipidemia, who was admitted with ALF potentially secondary to DILI. Prior to admission, he was receiving therapy that may be related to idiosyncratic DILI (I-DILI) and ALF, namely nintedanib, which appears to have a most probable relation to I-DILI in this case, considering it was the most recently started drug. Herpes simplex virus (HSV) type 1 was also identified and probably potentiated I-DILI development and ALF. Considering the patient's history and previous medical status, he was not considered eligible for liver transplantation in the setting of a multidisciplinary discussion. Despite therapy with N-acetylcysteine and acyclovir, there was progressive clinical deterioration with worsening of encephalopathy, and the patient died.

This case represents a rare and complex situation of ALF following I-DILI potentiated by HSV type 1 infection, reflecting its high mortality risk in patients not undergoing liver transplantation.

## Introduction

Acute liver failure (ALF) is a rare, life-threatening, and potentially reversible condition that occurs in patients without preexisting liver disease [1]. It is characterized by markers of liver injury (abnormal liver tests), coagulation disturbances with an international normalized ratio (INR) >1.5, and hepatic encephalopathy (HE) [1,2]. The most used definition of ALF implies an illness duration of less than 26 weeks [1]. In developed countries, an incidence of one to six cases of ALF per million population is estimated [1]. The etiology of ALF is an indicator of treatment strategies and prognosis [1,2]. Several etiologies of ALF have been described: drug-induced liver injury (DILI), including from acetaminophen hepatotoxicity or other drugs; viral hepatitis; pregnancy-related, namely elevated liver enzymes and low platelet (HELLP) syndrome and acute fatty liver of pregnancy; autoimmune hepatitis; Wilson disease; mushroom poisoning; Budd-Chiari syndrome; ischemic liver injury and malignant infiltration [1,2].

DILI represents the most common cause of ALF in the Western world despite its low incidence of 14-19 cases per 100,000 population [3,4]. Excluding acetaminophen toxicity, most cases of DILI are idiosyncratic in nature [3]. Idiosyncratic DILI (I-DILI) is unexpected based on the pharmacological actions of the drug, follows an unpredictable course with a highly variable latency period and clinical presentation, and lacks a clear dose dependence [1,4]. Early discontinuation of the offending agent is essential to prevent clinical deterioration, and a high clinical suspicion is needed to make the diagnosis. ALF secondary to I-DILI arises more often in those above 60 years and has a high mortality rate [2,4].

Some viral infections have been identified as causes of ALF or as co-factors for its development, namely herpes simplex virus (HSV) types 1 and 2, varicella-zoster virus (VZV), cytomegalovirus (CMV), and Epstein-Barr virus (EBV) [1,2]. These infections are most often implicated in cases of ALF in immunosuppressed individuals [1]. HSV infection may lead to viral hepatitis that can progress to ALF or may potentiate liver injury from other causes through mechanisms not completely understood.

We present a complex and rare case of a 73-year-old immunosuppressed patient admitted with ALF potentially associated with I-DILI and HSV infection. It highlights the importance of early recognition and prompt identification of causative factors that can be treated or removed, as a delayed diagnosis and directed approach can significantly increase mortality risk.

## Case presentation

A 73-year-old male presented to an emergency department with asthenia and progressively worsening dyspnea for about a month and a half. He also reported mild memory failures and insomnia during the week before admission.

The patient had a previous medical history of fibrotic hypersensitivity pneumonitis with a progressive phenotype requiring long-term oxygen therapy, for which he was receiving prednisone and azathioprine for approximately two years and antifibrotic therapy with nintedanib for almost a year with no documented side effects during follow-up. He also had type 2 diabetes mellitus for which he was medicated with vildagliptin, gliclazide, and empagliflozin; hypertension and hyperlipidemia treated with ramipril and rosuvastatin in combination with ezetimibe, respectively. In addition, he was also receiving omeprazole and glucosamine sulfate.

**Figure 1:**
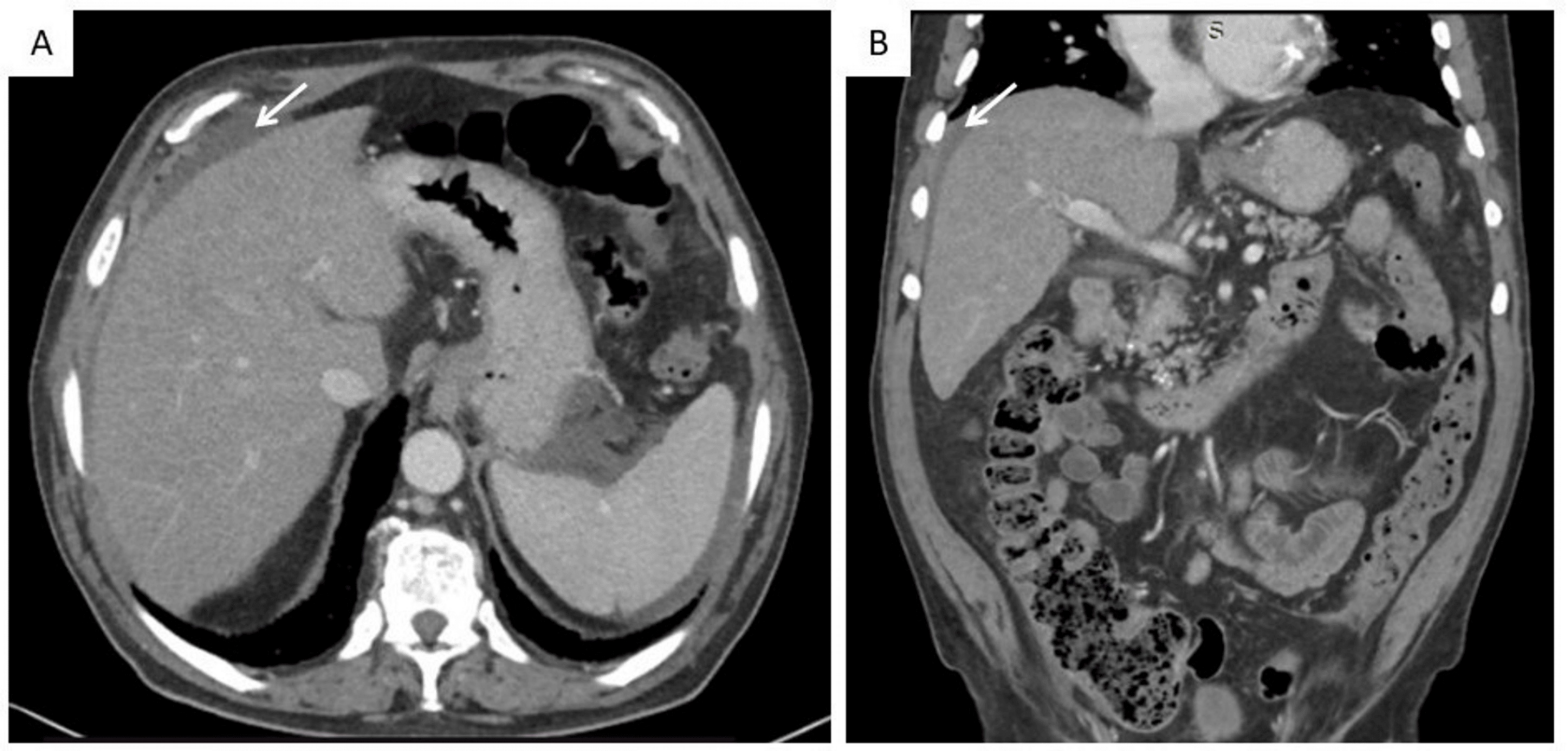
Thoraco-abdominopelvic computed tomography without signs of chronic liver disease but with small volume perihepatic ascites (white arrows) A) Axial plane; B) Coronal plane

Nine days earlier, he had gone to another emergency department where blood tests were carried out, revealing platelets 108 x 10^9^/L; total bilirubin level 1.0 mg/dL; aspartate aminotransferase (AST) 136 U/L; alanine transaminase (ALT) 136 U/L; alkaline phosphatase (ALP) 1699 U/L; gamma-glutamyl transferase (GGT) 730 U/L; lactate dehydrogenase (LDH) 2995 U/L; C-reactive protein (CRP) 56.4 mg/L. He also underwent a thoracic-abdominopelvic computed tomography (CT) with intravenous contrast that revealed the known findings related to the fibrotic hypersensitivity pneumonitis and small volume perihepatic ascites; no other findings were reported (Figure 1).

No other clinically relevant findings were noted, and the patient was discharged, maintaining the same therapy regimen, with information about signs to be aware of. Faced with worsening symptoms, as mentioned above, he went to a hospital again.

Upon his second admission, he was conscious, alert, oriented, without asterixis, and scored 15 points on the Glasgow Coma Scale; jaundiced; normothermic; hemodynamically stable; polypneic with a respiratory rate (RR) of 20-24 cycles/min, saturating at 98% with oxygen therapy by nasal cannula (one liter/ minute); on lung auscultation, rough breathing sound and crackles were audible bilaterally; abdomen examination as well as the remainder physical examination was unremarkable.

**Figure 2:**
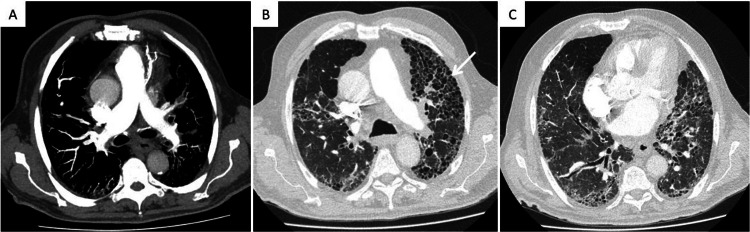
Chest computed tomography angiography excluding pulmonary embolism (A). Parenchymal changes in relation to fibrotic hypersensitivity pneumonitis (B, C), with a honeycomb pattern (white arrow)

Blood tests were remarkable for worsening: platelets- 84 x 10^9^/L; INR- 1.52; D-dimer- 48487 µg/L; factor V- 87.9%; total bilirubin- 3.24 mg/dL; direct bilirubin- 1.98 mg/dL; AST- 328 U/L; ALT- 163 U/L; ALP- 1771 U/L; GGT- 1082 U/L; LDH- 4226 U/L; CRP- 134.5 mg/L; lactate- 6.7 mmol/L. Considering the marked elevation of D-dimer, pulmonary embolism was excluded through chest CT-angiography (Figure 2).

ALF was suspected, and considering the revised history, the most probable etiology at this time was DILI. The patient denied a history of liver disease, alcohol use, or supratherapeutic acetaminophen ingestion. Immediately, all outpatient therapy was discontinued except prednisone, and fluid therapy was started in a controlled way, avoiding volume overload.

**Figure 3:**
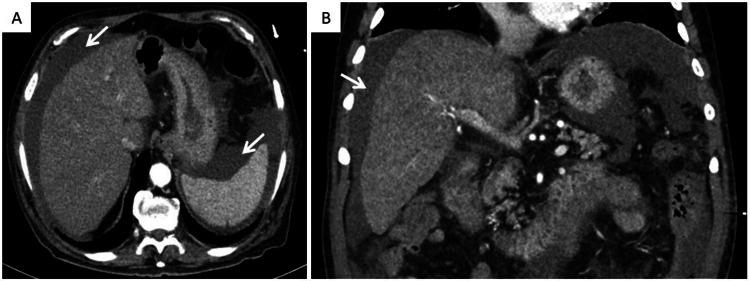
Abdominal computed tomography angiography excluding signs of chronic liver disease, Budd-Chiari syndrome, or malignant infiltration but revealing moderate ascites (white arrows). A) Axial plane; B) Coronal plane

An abdominal CT-angiography was also performed, excluding signs of chronic liver disease and causes of ALF, such as Budd-Chiari syndrome or malignant infiltration, only revealing moderate ascites (Figure 3).

The case was discussed with the multidisciplinary transplant team within the first hours of admission regarding liver transplant candidacy, and, considering the patient's age and previous medical status with severe fibrotic lung disease and respiratory failure, he was not considered eligible.

According to the main hypothesis of ALF secondary to I-DILI, intravenous N-acetylcysteine (NAC) protocol was initiated on the first day of hospitalization. Rifaximine and lactulose were also initiated for HE, and monitoring and correction of fluid and electrolyte imbalances were maintained. Despite this, there was progressive clinical worsening, with HE progressing to grade 2 on the third day. The case was then discussed again in a multidisciplinary team, including intensive care, to determine whether the patient would benefit from renal replacement therapy to manage hyperammonemia. After careful evaluation, considering the global prognosis, it was agreed that there would be no benefit. Correction of coagulopathy was not performed as there were no signs of active bleeding. Extended NAC protocol was continued. Blood and urine cultures were used to exclude concomitant infections, and the results were negative.

Other causes of ALF, such as viral and autoimmune hepatitis, were studied. Screening for hepatitis A, B, C, and E was negative; screening for VZV, CMV, and EBV was also negative. Antinuclear antibodies, anti-smooth muscle actin antibodies, and immunoglobulins were normal. On the fifth day of hospitalization, a positive result for HSV type 1 was obtained by polymerase chain reaction (PCR), and antiviral therapy with intravenous acyclovir was started, considering HSV infection can potentiate or cause ALF.

Unfortunately, the patient presented progressive clinical and laboratory worsening (Table 1). On the sixth day of hospitalization, HE progressed to grade 3, and on the seventh day, multiorgan failure ensued, and the patient died.

**Table 1:** Laboratory parameters evolution during hospitalization Abbreviations: INR: International normalized ratio; AST: Aspartate aminotransferase; ALT: Alanine transaminase; GGT: Gamma-glutamyl transferase; ALP: Alkaline phosphatase; LDH: lactate dehydrogenase; CRP: C-reactive protein

Laboratory parameters	Day 1	Day 2	Day 3	Day 4	Day 5	Day 6	Reference range
Hemoglobin (x10g/L)	14.0	13.4	13.7	14.1	14.5	14.0	13.0-17.0
Leukocytes (x10^9^/L)	5.15	4.28	3.81	4.93	4.98	5.66	4.5-11.0
Neutrophil (x10^9^/L)	3.82	3.24	2.70	3.36	3.54	3.76	2.0-8.5
Lymphocyte (x10^9^/L)	0.83	0.57	0.75	1.14	0.91	1.14	0.9-3.5
Platelets (x10^9^/L)	84	63	64	71	72	55	150-450
INR	1.52	1.58	1.8	1.88	2.18	2.83	0.80-1.20
Fibrinogen (g/L)	1.4	1.5	1.2	1.2	1.1	0.8	2.00-4.00
D-dimer (μg/L)	48487	40132	48296	47684	43543	-	<500
Factor V (%)	87.9	-	74.6	73.1	55.2	39.8	62-139
Urea (mg/dL)	69	73	62	69	84	119	16.6-48.5
Creatinine (mg/dL)	0.93	0.82	0.73	1.09	0.98	1.35	0.67-1.17
Total bilirubin (mg/dL)	3.24	3.66	3.91	5.42	6.18	8.06	<1.40
Direct bilirubin (mg/dL)	1.98	2.57	2.68	3.5	3.87	5.58	<0.50
AST (U/L)	328	359	359	406	305	351	<40
ALT (U/L)	163	161	168	184	166	174	<41
GGT (U/L)	1082	1096	1150	1190	1197	1099	<60
ALP (U/L)	1771	1663	1907	1738	1756	1993	40-129
LDH (U/L)	4226	4106	4663	4827	4923	5472	135-225
Amylase (U/L)	33	26	-	-	-	-	13.00-53.0
Ammonia (μg/dL)	-	104	-	230	219	279	18.7-86.9
CRP (mg/L)	134.5	112.0	103.1	92.8	93.9	102	<5.0
Lactate (mmol/L)	6.7	6.8	6.2	9.5	-	-	0.5-1.6

## Discussion

ALF requires prompt recognition and a targeted approach, considering its high mortality rate in those not undergoing liver transplantation. Defining its etiology is essential to define treatment strategies. In the case presented, ALF was considered secondary to I-DILI and potentiated by HSV infection once other causes of ALF were excluded. This patient was being treated with some pharmacological drugs that have been reported as related to some cases of I-DILI and ALF.

ALF associated with I-DILI usually has an acute-to-subacute presentation and is not necessarily dose-dependent. Although I-DILI generally has a favorable prognosis, those progressing to ALF have transplant-free survival of 24-39% at 3 weeks and an overall survival rate of 66% [1].

Albeit rare, nintedanib-associated DILI has emerged as an adverse event in clinical trials [5]. A recent retrospective cohort study using a nationwide health insurance database explored DILI in patients with idiopathic pulmonary fibrosis and confirmed the increased risk of DILI in those treated with nintedanib during the first year of treatment [6]. According to reported cases, presentation is variable, with a latency to onset from 4 to 24 months and a pattern of enzyme elevations from cholestatic to hepatocellular [7]. Fatal cases have been reported, but causality is often only possible or, at most, probable [7].

Azathioprine has also been associated with different I-DILI, which usually presents with a cholestatic pattern within the first year of therapy, although there are cases reported multiple years after azathioprine initiation [8,9]. The liver injury usually resolves rapidly once azathioprine is stopped, but some prolonged and fatal cases have been reported [9].

Clinically apparent liver injury from the second generation sulfonylureas is infrequent but has been reported and appears within 3 to 12 weeks of starting the medication, although rare cases are reported later [10]. Sulfonylureas are rarely listed as causes of ALF [10].

Of the remaining therapy the patient received prior to hospitalization, ramipril, rosuvastatin, ezetimibe, and omeprazole may also be associated with serum aminotransferase elevations but usually during the first months of treatment and rarely with clinically apparent acute liver injury [11-14]. Glucosamine has been related to acute liver injury in isolated case reports and very rarely with ALF, but the role of glucosamine as opposed to other herbal components has not been determined [15].

In this case, it is difficult to determine which drug could be related to ALF, but considering that nintedanib was the most recently introduced drug, it appears to have the most probable relation.

As performed currently, when ALF secondary to I-DILI is suspected, the causative drug should be discontinued immediately, and subsequent care is mostly supportive [1,2]. Although no specific therapy is approved for the treatment of I-DILI, intravenous NAC is recommended, according to studies that demonstrate it increases survival [1].

HE is a main manifestation of ALF and should be closely monitored [2]. Unfortunately, in this scenario, the use of lactulose or rifaximin has no evidence [1,2]. Continuous renal replacement therapy (CRRT) has been shown to effectively lower ammonia levels in ALF patients, and early CRRT is suggested to manage hyperammonemia [1]. In this case, CRRT was not performed according to multidisciplinary discussions, considering the patient's previous condition and overall prognosis given pulmonary disease, but it should always be considered.

Another key factor is HSV infection. HSV hepatitis is an uncommon cause of ALF, usually not identified until after liver transplantation or death, and the limited cases reported occurred more frequently in immunosuppressed patients [16,17]. Although HSV infection might rarely represent the etiology of the ALF, it may be a co-factor and potentiate the development of DILI [2]. In less than half of the reported cases, mucocutaneous lesions suggestive of HSV were present [16]. Occult HSV screening using PCR for HSV DNA should be performed when ALF etiology is uncertain [2,17]. In this case, as the patient was immunosuppressed, PCR for HSV DNA was performed, but as there was no high clinical suspicion for HSV infection and there were other potential ALF causes, treatment was initiated only after HSV 1 detection. Recent guidelines recommend empiric treatment with acyclovir in ALF patients with grade 2 HE and features suggestive of HSV or zoster infection until confirmatory testing is obtained but do not recommend empiric treatment in patients without HSV infection suspicion [1]. This case highlights this topic and may suggest that antiviral empiric treatment should be considered in all immunosuppressed patients with ALF. In many of the cases reported patients with HSV hepatitis presented with an anicteric pattern [16]. This patient was icteric, which suggests that HSV infection was essentially a potentiator rather than the cause of ALF.

## Conclusions

I-DILI can progress to ALF, representing a challenging diagnosis that implies high clinical suspicion and an early approach. This case highlights the importance of regularly monitoring patients on certain medications that are associated with DILI as well as the importance of early withdrawal of that drug, which can seriously influence a patient's prognosis. Furthermore, it regards a rare situation of an ALF possibly due to nintedanib. Systematic reporting of these situations should be performed. Also noteworthy is the importance of excluding HSV infection, especially in immunosuppressed patients, even in the presence of another possible etiology for ALF, since it can be a co-factor and potentiate ALF progression, requiring early antiviral therapy.

The case presented, unfortunately, reflects the high mortality risk in patients with ALF following I-DILI and HSV infection, not undergoing liver transplantation. Further investigation is needed regarding developing treatment strategies for these patients.
